# Examining opioid agonist treatment (OAT) site operations and early signals of change in the first year of British Columbia’s drug decriminalization policy: Insights from a provincial survey

**DOI:** 10.17269/s41997-025-01060-2

**Published:** 2025-06-09

**Authors:** Cayley Russell, Justine Law, Kate Hodgson, Laura MacKinnon, Rita Shahin, Frank Crichlow, Sean Patenaude, Sameer Imtiaz, Jürgen Rehm, Farihah Ali

**Affiliations:** 1 Institute for Mental Health Policy Research (IMHPR), Centre for Addiction and Mental Health (CAMH), Toronto, ON Canada; 2 Ontario Node, Canadian Research Initiative in Substance Matters (CRISM), Toronto, ON Canada; 3 Department of Nurse Practitioners, Vancouver Coastal Health, Vancouver, BC Canada; 4 Harm Reduction Nurses Association, University of Victoria, Victoria, BC Canada; 5 University of British Columbia (UBC), First Nations Health Authority (FNHA), Vancouver, BC Canada; 6 Toronto Public Health, Toronto, ON Canada; 7 Canadian Association of People Who Use Drugs (CAPUD), Dartmouth, NS Canada; 8 Department of Patient and Family Experience, Centre for Addiction and Mental Health (CAMH), Toronto, ON Canada; 9 Dalla Lana School of Public Health, University of Toronto, Toronto, ON Canada; 10 Department of Psychiatry and Institute for Medical Sciences, Temerty Faculty of Medicine, University of Toronto, Toronto, ON Canada; 11 Campbell Family Mental Health Research Institute, Centre for Addiction and Mental Health (CAMH), Toronto, ON Canada; 12 Program on Substance Abuse & WHO European Region Collaborating Centre, Public Health Institute of Catalonia, Barcelona, Spain; 13 Center for Interdisciplinary Addiction Research (ZIS), Department of Psychiatry and Psychotherapy, University Medical Center Hamburg-Eppendorf (UKE), Hamburg, Germany

**Keywords:** Decriminalization, Drug policy, Opioid agonist treatment (OAT), People who use drugs, Pharmacotherapy, Public health, Décriminalisation, Politique antidrogue, Traitement par agonistes opioïdes (TAO), Personnes faisant usage de drogue, Pharmacothérapie, Santé publique

## Abstract

**Objective:**

On January 31, 2023, the province of British Columbia (BC) introduced a 3-year drug decriminalization initiative, with a goal of increasing access, engagement, and retention in drug use treatment. There is limited information on the operational characteristics of opioid agonist treatment (OAT) sites in BC. These data are required to monitor the impacts of decriminalization on these outcomes. This study sought to characterize OAT service operations and examine any preliminary operational changes following decriminalization, from the perspectives of OAT site staff.

**Methods:**

Between March and April 2024, a cross-sectional, online self-report survey was distributed to OAT sites across BC, completed by site representatives. Questions focused on OAT service operations, including service capacity, treatment retention, and clientele demographics, as well as potential changes to service operations due to decriminalization. Data were analyzed descriptively.

**Results:**

A total of 28 OAT sites from across BC completed the survey. Findings suggest that decriminalization has had limited impacts on OAT site operations within the first year of the policy’s implementation. However, several sites reported early signals of change related to client socio-demographics, including seeing more male and younger clients, as well as an increase in demand on their staff and resources.

**Conclusion:**

Despite minimal changes to OAT site operations within the first year of BC’s decriminalization policy, findings suggest the need for increased staff training on decriminalization and continued investments into OAT to better support the anticipated demand on services if decriminalization is to successfully reach its goal of improving access to treatment.

## Résumé

**Objectif:**

Le 31 janvier 2023, la province de la Colombie-Britannique a introduit une initiative triennale de décriminalisation des drogues dans le but d’améliorer l’accès et la participation aux traitements de l’usage de drogues et la rétention en traitement. Il existe peu de données sur les caractéristiques opérationnelles des établissements de traitement par agonistes opioïdes (TAO) en Colombie-Britannique, alors qu’elles sont nécessaires pour observer les effets de la décriminalisation sur les résultats cliniques. Nous avons voulu caractériser le fonctionnement des services de TAO et examiner les changements opérationnels préliminaires apportés depuis la décriminalisation du point de vue du personnel des établissements de TAO.

**Méthode:**

En mars et avril 2024, nous avons diffusé en ligne un questionnaire transversal d’autoévaluation aux établissements de TAO de la Colombie-Britannique, qui ont été remplis par leurs représentants. Les questions portaient sur le fonctionnement des services de TAO, dont la capacité des services, la rétention en traitement et les données démographiques de la clientèle, et sur les éventuels changements aux services découlant de la décriminalisation. Nous avons procédé à l’analyse descriptive des données.

**Résultats:**

Vingt-huit établissements de TAO de la province ont envoyé des réponses au questionnaire. Leurs réponses indiquent que la décriminalisation a eu peu d’effets sur le fonctionnement des établissements de TAO au cours de l’année qui a suivi la mise en œuvre de la politique. Plusieurs établissements ont cependant fait état de premiers signes de changements dans le profil sociodémographique de leur clientèle, dont la présence de plus d’hommes et de jeunes, et d’une augmentation de la demande en personnel et en ressources.

**Conclusion:**

Bien qu’il y ait eu peu de changements dans le fonctionnement des établissements de TAO au cours de l’année qui a suivi la politique de décriminalisation en Colombie-Britannique, nos constats indiquent le besoin d’accroître la formation du personnel sur la décriminalisation et de continuer d’investir dans le TAO pour mieux soutenir la demande de services anticipée si la décriminalisation atteint son objectif d’améliorer l’accès au traitement.

## Introduction

Over the past decade, Canada has seen a substantial increase in opioid-related deaths and harms, which are now driven primarily by fentanyl and the unregulated toxic drug supply (Government of Canada, 2024). Between January 2016 and December 2023, there were a total of 44,592 apparent opioid toxicity deaths, and 42,711 opioid-related poisoning hospitalizations nationwide, many of which also involved stimulants (Government of Canada, 2024). The province of British Columbia (BC), where the overdose crisis was declared a public health emergency in 2016, has consistently faced higher rates of overdose deaths and harms compared to other provinces and to Canada as a whole (e.g., 46.6/100,000 overdose deaths in BC versus 20.8/100,000 in Canada) (Government of Canada, 2024). Despite this, BC has a long history of pioneering treatment and prevention initiatives to address drug use–related harms, including becoming the first province to introduce opioid agonist therapy (OAT) in 1963 (Boyd, 2021).

OAT is the gold standard pharmacotherapy treatment for opioid use disorder (OUD) in Canada, involving the use of prescribed opioid agonist medications to prevent withdrawal and reduce cravings (Yakovenko et al., 2024). Updated 2024 guidance suggests that methadone and buprenorphine/naloxone (Suboxone) are both recommended as first-line OAT medications. Despite not being formally approved for the treatment of OUD in Canada, slow-release oral morphine (SROM) is recommended as a second-line medication, and is commonly prescribed for this use off-label, depending on the prescriber (Yakovenko et al., 2024). Other OAT medications are also available, such as injectable extended-release buprenorphine (Sublocade), as well as injectable diacetylmorphine and injectable hydromorphone; however, these are less commonly prescribed, with the latter two often reserved for those with severe OUD and ongoing unregulated injection opioid use who have not benefited from traditional oral OAT options (British Columbia Centre on Substance Use (BCCSU), 2023). These medications, despite their varying pharmacokinetics and pharmacodynamics, share similar clinical delivery practices. In BC, OAT medications are generally provided within private and provincially funded specialty addiction clinics (including rapid access addiction clinics), or through general practice or primary care clinics (Eibl et al., 2017).

Typically, patients begin OAT with witnessed medication doses by a healthcare practitioner. After a period of consistent treatment to establish medication stability, guidelines suggest that patients may be eligible for unwitnessed or take-home doses, which allow people who use drugs (PWUD) the flexibility to integrate their treatment into their daily lives (Russell et al., 2024). However, the provision of take-home doses is often contingent on demonstrating clinical and social stability, which can be difficult for many PWUD and can result in disparities regarding who is eligible to receive take-home doses (Russell et al., 2024).

Since the declaration of the public health emergency, BC has seen an increase in OAT uptake, with the number of individuals dispensed OAT increasing from 14,743 in 2015 to 24,232 in 2023 (BC Centre for Disease Control (BCCDC), 2023). OAT services have a well-documented beneficial impact on the health of PWUD (Pearce et al., 2020). Long-term engagement in OAT has been shown to significantly benefit PWUD and has been linked to decreases in illicit drug use, hospitalizations, criminality, mortality rates, and healthcare costs, as well as improvements in quality of life for PWUD (Socías et al., 2020).

Despite these benefits, long-term retention in OAT remains low (Socías et al., 2020). Barriers such as social and structural factors related to stigma, criminalization of substance use, and rigorous program requirements (e.g., frequent clinic visits) hinder engagement and retention in OAT (Pijl et al., 2022; Socías et al., 2020). Confidentiality concerns in smaller communities also contribute to stigma, with PWUD expressing apprehension about others becoming aware of their drug use (Hodgson et al., 2024). Furthermore, the increasingly toxic drug supply in BC, contaminated with potent opioid (e.g., fentanyl) and non-opioid substances (e.g., benzodiazepines), poses additional challenges (Russell et al., 2023b). Moreover, there has been a rise in polysubstance use and associated harms (e.g., overdose), with more PWUD reporting using opioids combined with stimulants (methamphetamine, cocaine) (Dong et al., 2020). These changes have introduced new complexities to OAT treatment efforts, as PWUD may require higher OAT doses or find it insufficient to meet their needs, leading to treatment attrition (Russell et al., 2023b; Socías et al., 2020). For instance, concurrent methamphetamine use among individuals engaged in OAT is associated with an increased risk of treatment discontinuation and requires providers to address concurrent opioid and stimulant use in their clinical approach (Cui et al., 2022).

In response to this evolving crisis, BC piloted a 3-year drug decriminalization policy, commencing on January 31, 2023, under an exemption to Canada’s drug policy statute, *The Controlled Drugs and Substances Act* (Ministry of Mental Health & Addictions, 2023). This policy eliminates criminal penalties (e.g., arrests, fines, or confiscation of drugs) for the possession of up to 2.5 g of opioids, methamphetamine, cocaine/crack-cocaine, and MDMA/ecstasy for personal use among adults (Ministry of Mental Health & Addictions, 2023). This initiative represents a significant shift from traditional punitive approaches, focusing on reducing stigma and fear of criminalization as a means to increase access to treatment for PWUD, with the ultimate goal of addressing the toxic drug crisis in BC. In particular, the policy explicitly states several short- and long-term goals related to increasing engagement and retention in treatment services (Ministry of Mental Health & Addictions, 2023). For instance, short-term goals include increasing awareness and comfort in accessing health and social services among PWUD, while longer-term goals include reducing barriers to accessing health services and increasing engagement in treatment for PWUD. Crucially, however, the policy also states that any measurable progress towards these goals will rely on complementary health system changes and investments (e.g., expansion of harm reduction and treatment programs and improvements in health service delivery).

As such, ongoing monitoring and evaluation of the policy’s outcomes throughout its implementation are crucial to evaluate its effectiveness in meeting these goals. However, there are limited data on the current state of OAT programs in BC, including specifically in relation to site operations. These data are important not only to understand the landscape of OAT programs across the province, but also to be able to identify whether and how operations may change in light of the decriminalization policy. The purpose of this study was therefore twofold: (1) to characterize the current operational landscape of OAT service provision in BC, including specifically related to site settings, clientele profiles, staffing, OAT medications provided, treatment engagement and retention, referral pathways, and community impacts, and (2) to examine any early signals of change towards reaching the policy’s stated short- and long-term goals. This paper presents the findings from the first year of a multi-year longitudinal evaluation examining potential changes to OAT service operations following the implementation of BC’s decriminalization policy, provided by OAT site representatives. The results of this study can provide valuable insights on the current landscape of OAT sites in BC, as well as how these indicators may have changed in response to decriminalization thus far. These findings can serve as a baseline for monitoring the effects of the decriminalization policy on OAT provision and uptake in BC going forward.

## Methods

### Study design

We conducted a cross-sectional study of OAT site operations across BC using an online, self-administered survey distributed to OAT site representatives who had operational knowledge of the site (such as directors, program managers, or intake workers). We aimed to have one representative from each OAT-eligible site complete the survey, enabling us to gather comprehensive information on OAT services across the province.

### Eligibility

We included all standalone OAT clinics (both private and public) whose primary purpose was addiction treatment. Additionally, we included harm reduction sites—such as overdose prevention or supervised consumption services—and rapid access to addiction medicine/care (RAAM/RAAC) clinics that had integrated OAT services. These site types were selected because they primarily serve individuals with complex substance use needs, offer low-barrier access, provide more flexible dosing, and have staff with specialized expertise in substance use care.

We excluded broader community health centres/clinics, health units, and pharmacies that distribute OAT medications, as well as general practitioners or physicians who prescribe OAT through primary or emergency care settings, as their primary focus is not addiction treatment. This approach ensured that our study focused on sites most likely to experience direct impacts from drug decriminalization.

Since the decriminalization policy only applies to adults 18 years and older, we also excluded sites catering specifically to youth (i.e., those serving individuals under 18 or with a youth-specific focus). In addition, OAT sites that opened after the implementation of the decriminalization policy were excluded, as they could not provide insight into operational changes resulting from the policy.

We did not impose specific eligibility criteria for survey respondents. However, we encouraged each site to designate a respondent with direct knowledge of site or organizational operations. This approach ensured that survey responses reflected accurate and informed perspectives on site functionality, service delivery, and any changes experienced following drug decriminalization.

### Participant recruitment

We employed a multi-pronged recruitment strategy to identify OAT sites that met our inclusion criteria, as well as the most suitable OAT site representatives to respond to our survey. Initially, we compiled a comprehensive list of all OAT services across BC using established online repositories (e.g., “Toward the Heart”), resulting in approximately 62 potentially eligible harm reduction and OAT sites. The list was then categorized by the five regional health authorities in BC—Fraser Health, Vancouver Coastal Health, Interior Health, Island Health, and Northern Health—and was subsequently shared with our research network, including an established project working group consisting of people with lived and living experience with drug use and experts in the field. We also collaborated closely with several groups, including the Decriminalization Leads from each of the five regional health authorities and the BC Centre on Disease Control (BCCDC) Harm Reduction Coordinator team. Our collaborators helped identify any missing or ineligible sites, as well as site representatives best suited to respond to the survey. For sites where our contacts were not able to identify a representative, we conducted independent searches online and made telephone calls to directly identify site representatives. The final site list included 32 eligible sites.

### Measures

The survey included both quantitative and open-ended questions, developed in collaboration with the research team’s working group. The final survey was developed and distributed using Research Electronic Data Capture (REDCap), a secure web application for building and managing online surveys. The questions focused on various aspects of OAT services, including service capacity, prescriber information, waitlists, eligibility criteria, types of OAT offered, access to and provision of OAT take-home doses, service utilization/uptake, treatment retention, resources (staffing, infrastructure), referral pathways, and clientele sociodemographics. Each question included a follow-up asking whether decriminalization had impacted this aspect of their service, measured using yes/no/I don’t know response options. For all “yes” responses, questions further probed respondents to provide an open-ended response describing the changes experienced.

### Data collection

To maximize responsiveness and ensure comprehensive data collection, survey dissemination was strategically coordinated. Our contacts within the five regional health authorities and the BCCDC forwarded emails including information on the purpose of the survey, site eligibility information, and a link to the consent form and survey. For sites where we independently identified a representative, we distributed the survey invitation link by inputting the site representative’s email directly into the REDCap platform and enabling automatic emails. We set the length of the survey fielding a priori at approximately two months*,* specifying that we would send an initial invitation, followed by two reminder emails sent approximately once every two weeks. Survey invitations were sent out on a rolling basis. The initial survey was launched on March 15, 2024, with additional invitations sent out through April 10, 2024. Data collection concluded on May 31, 2024.

### Consent, remuneration, and ethics

Prior to beginning the survey, participants were required to read and sign an informed consent form embedded into REDCap, outlining their rights as participants and additional study details. The consent form assured participants that all responses would be de-identified and confidential. All participants were provided the opportunity to opt in to receive a $25 Amazon e-gift card for completing the survey by providing their contact information, which was collected and stored in a separate form.

This study is part of an independent evaluation of BC’s decriminalization policy being carried out by the Ontario Node of the Canadian Research Initiative in Substance Matters (CRISM), housed within the Centre for Addiction and Mental Health (CAMH) in Toronto, Ontario. This study was approved by CAMH’s research ethics board (REB#2023/169).

### Data analysis

Data were exported from REDCap to an Excel database, cleaned, and subsequently imported into SPSS. Analyses consisted of basic descriptive statistics, including frequency distributions and percentages. There were four missing responses, which were excluded from our analyses. In cases where data were missing, or follow-up questions did not apply to the respondent, we included the denominator to highlight the number of sites included in those specific calculations. We classified the community each site was located in as either rural or urban, using Statistics Canada’s Index of Remoteness (RI) (Statistics Canada, 2023). Sites from communities with an RI score of less than 0.29 were classified as rural, while sites from communities with RI scores greater than 0.29 were classified as urban.

## Results

A total of 32 OAT services were invited to participate in the survey, including three services that were identified through referrals from affiliated service contacts. Two survey responses were excluded due to duplication from the same site (with only the first response retained), and one additional survey response was excluded because the site opened post-decriminalization. In total, 28 OAT sites completed the survey, yielding an 88% response rate.

### Survey respondent role characteristics

The OAT site representatives who completed the survey held various clinical and administrative roles, including program managers (*n* = 6/28; 21%), supervisors (*n* = 5/28;18%), intake/administration (*n* = 3/28; 11%), registered nurses (*n* = 3/28; 11%), site directors (*n* = 2/28; 7%), and physicians (*n* = 1/28; 4%). Additionally, other roles included clinical leads and support and outreach workers (*n* = 8/28; 29%). Many participants (*n* = 13/28; 46%) had been in their roles for more than 5 years, while nearly a third (*n* = 9/28; 32%) had been employed for between 1 and 5 years. The remaining participants (*n* = 6/28; 21%) had been in their roles for less than a year.

### OAT site characteristics

#### Location

The 28 OAT sites were distributed across BC’s five health authority regions, including Fraser Health Authority (*n* = 10/28; 36%), Interior Health Authority (*n* = 5/28; 18%), Island Health Authority (*n* = 3/28; 11%), Northern Health Authority (*n* = 3/28; 11%), and Vancouver Coastal Health Authority (*n* = 7/28; 25%). The majority (20/28; 71%) of OAT sites were located in urban communities, of which half (*n* = 10/20; 50%) were located in the Fraser Health Authority region, six (*n* = 6/20; 30%) were in the Vancouver Coastal Health Authority region, three (*n* = 3/20; 15%) were in the Island Health Authority, and one (*n* = 1/20; 5%) was located in the Interior Health Region. The remaining eight (8/28; 28%) sites were located in rural/remote communities within the Interior Health Authority (*n* = 4/8; 50%), Northern Health Authority (*n* = 3/8; 38%), and Vancouver Coastal Health Authority (*n* = 1/8; 13%).

#### OAT site establishment and setting

Among the 28 OAT sites, four (*n* = 4/28; 14%) were established in 1999, just over a fifth (*n* = 6/28; 21%) in 2018, and another four (*n* = 4/28; 14%) in 2021. The remaining 14 sites (50%) were established at varying times between 1999 and 2021. Nearly half of the OAT sites (*n* = 12/28; 43%) were fully integrated within a broader organization offering additional health or social services (i.e., located on-site). Close to a third (*n* = 8/28; 29%) were affiliated with another organization but operated independently, such as a satellite or mobile site.

#### OAT site operations

Most OAT sites (*n* = 25/28; 89%) reported being open at least five days per week, with seven sites (*n* = 7/28; 25%) open daily. One site operated four days a week, while two sites (*n* = 2/28; 7%) were only open one day a week. Eight sites (*n* = 8/28; 28%) offered services on Saturdays, and six sites (*n* = 6/28; 21%) were open on Sundays. Additionally, one site (*n* = 1/28; 4%) operated on Fridays for only three weeks each month. On average, sites were open for 8.3 hours per day (standard deviation (SD) 0.47).

The majority of sites (*n* = 24/28; 86%) reported no changes to their hours of operation since decriminalization. However, one site (*n* = 1/28; 4%) extended its hours, and two sites (*n* = 2/28; 7%) increased the number of days they were open.

#### OAT site staffing, training, and resources

Physicians, nurse practitioners, and registered nurses all prescribed OAT for clients. Most sites (*n* = 25/28; 89%) employed a physician, while almost a third of sites (*n* = 9/28; 32%) employed registered nurses, and five sites (*n* = 5/28; 18%) employed nurse practitioners. One respondent (*n* = 1/28; 4%) indicated that they did not know the staffing structure at their site. Two sites (*n* = 2/28; 7%) did not have prescribers on payroll, but contracted these positions externally.

Over a third (*n* = 11/28; 39%) of OAT sites were staffed by multidisciplinary and comprehensive teams consisting of administrative personnel, OAT prescribers (physicians, registered nurses, or nurse practitioners), and broader health and social support workers (e.g., counsellors/therapists, social workers, peer support providers, outreach workers, liaisons, and infectious disease workers). The remaining sites had various staff combinations comprised of these roles, and four sites (*n* = 4/28; 14%) had a pharmacist on staff who dispensed OAT medications.

Most sites (*n* = 18/28; 64%) indicated that their current staffing levels were sufficient to meet client needs. However, eight sites (*n* = 8/28; 29%) reported that staffing was insufficient to meet service demands. Six programs (*n* = 6/28; 21%) reported changes in the demand on their staff since decriminalization, with all but one reporting an increase.

We also sought to determine whether OAT service personnel received any training specific to BC’s decriminalization initiative. Only three sites (*n* = 3/28; 11%) indicated that their staff received formal decriminalization training, which was delivered through online modules at all three sites. One of these programs also offered in-person training. Two of the three sites indicated that the training provided their staff with adequate knowledge of the decriminalization policy. Among the remaining sites that had not received formal decriminalization training, over a third (*n* = 9/24; 38%) indicated that their site/staff would benefit from this training.

In addition to staffing demands, survey respondents were also asked whether they felt their current resources (supplies, space, equipment) met their site’s demand. Responses were split, with just under half of the respondents (*n* = 13/28; 46%) reporting that their resources were adequate, while an equal number (*n* = 13/28; 46%) indicated that their resources were insufficient to meet their clients’ needs. Further, eight programs (*n* = 8/28; 29%) reported an increase in the demand on their resources post-decriminalization.

#### OAT medications offered

The types of OAT medications that were offered by the surveyed sites varied. All but one site (*n* = 27/28; 96%) offered both methadone and Suboxone, while 26 sites (*n* = 26/28; 93%) provided SROM. Other OAT medications offered at sites included Sublocade, transdermal/patch fentanyl, injectable liquid hydromorphone, and injectable tablet hydromorphone. Methadone was reported as the most frequently prescribed OAT medication at the majority of sites (*n* = 17/28; 61%), followed by Suboxone (*n* = 7/28; 25%), and SROM (*n* = 2/28; 7%). Table 1 provides an overview of the OAT medications prescribed at sites.

**Table 1:** Overview of OAT medications and take-home doses provided by OAT sites (*n* = 28)

	Sites	
	*n*	%
Medications offered by OAT sites		
Methadone	27	96
Buprenorphine/naloxone (Suboxone)	27	96
Slow-release oral morphine (SROM)	26	93
Injectable extended-release buprenorphine (Sublocade)	23	82
Transdermal/patch fentanyl	14	50
Injectable hydromorphone	7	25
Tablet injectable hydromorphone	6	21
Other safer supply medications	4	14
OAT take-home doses provided		
Yes	22	78
* Increased post-decriminalization*	*3*	*11*

The majority of OAT sites indicated that they offer OAT take-home doses (*n* = 22/28; 78%), three (*n* = 3/22; 14%) of which reported an increase in the frequency of take-home dose prescriptions following the implementation of decriminalization (Table 1).

### OAT clientele profiles

Most OAT sites (*n* = 24/28; 86%) did not have a specific clientele focus (e.g., women-identifying only, pregnant people/parents, Indigenous-serving, LGBTQ2S +, youth-specific) and accepted all clients. However, one site (*n* = 1/28; 4%) specialized in accepting patients experiencing homelessness, and another (*n* = 1/28; 4%) provided services specifically for clients living in the Downtown Eastside of Vancouver who wished to engage in OAT but were not connected to a healthcare provider. Demographic details of clientele, including age, gender, and ethnicity, are further outlined below.

#### Age

The majority of OAT sites (*n* = 20/28; 71%) served clients of all ages. Among the remaining sites, primary clientele ages varied: five sites (*n* = 5/28; 18%) primarily served clients aged 30 to 49 years, while one site (*n* = 1/28; 4%) mainly served younger clients aged 18 to 29, and another (*n* = 1/28; 4%) primarily served older clients, generally ranging from 50 to 69 years old.

Only one respondent (*n* = 1/28; 4%) noted a change in the average age of their primary clientele since decriminalization, observing an increase in youth under the age of 18 (and particularly those experiencing homelessness).

#### Gender

Nearly a third of sites (*n* = 9/28; 32%) reported serving an equal number of men, women, and gender-expansive clients. Four sites (*n* = 4/28; 14%) indicated they serve an equal number of men and women, but fewer gender-expansive individuals. Five sites (*n* = 5/28; 18%) primarily served clients who identify as men, while one site primarily served women-identifying clients, and another primarily served gender-expansive individuals.

Most respondents (*n* = 23/28; 82%) reported no changes in the gender profile of their primary clientele post-decriminalization. However, one respondent (*n* = 1/28; 4%) noted an increase in the number of men accessing their services.

#### Ethnicity

Among the surveyed OAT sites, the majority (*n* = 23/28; 82%) reported that their primary clientele were white, followed by Indigenous individuals (*n* = 3/28; 61%). One site (*n* = 1/28; 4%) primarily saw multiethnic clients.

None of the respondents reported observing any change in the ethnicity of their primary clientele since decriminalization came into effect.

### OAT service capacity, waitlists, and retention

The average number of clients engaged in OAT across sites varied significantly, ranging from fewer than 25 to over 300 clients per month. Specifically, half of the programs (*n* = 14/28; 50%) reported serving over 100 clients each month, while eight programs (*n* = 8/28; 29%) served an average of 50–100 clients per month. Four programs (*n* = 4/28; 14%) noted that fewer than 50 clients were engaged in OAT at their site in any given month.

Although the majority of sites (*n* = 16/28; 57%) did not experience a change in the average number of clients accessing their site since decriminalization, six respondents (*n* = 6/28; 21%) reported observing changes. Among these, three sites (*n* = 3/6; 50%) saw an increase in clients, and three (*n* = 3/6; 50%) saw a decrease in clients.

None of the OAT sites reported currently having a waitlist for clients to access OAT, and three quarters (*n* = 21/28; 75%) indicated that they offer same day/drop-in services. The average wait time for drop-in services varied, with over half of the sites (*n* = 12/21; 57%) reporting wait times of less than one hour. Nearly a third (*n* = 6/21; 29%) had average wait times of 1–2 hours, and one site (*n* = 1/28; 4%) reported that clients could usually receive OAT on the same day they drop in.

Respondents were also asked about the average length of time clients typically remained engaged in OAT at their site. Over a third of sites (*n* = 10/28; 34%) indicated the average length of treatment among clients was over a year, while four sites (*n* = 4/28; 14%) reported average engagement periods of six months to a year. One site (*n* = 1/28; 4%) indicated that clients remained engaged for one to three months. Six additional sites (*n* = 6/28; 21%) reported varying treatment retention times, ranging from one week to over ten years. A quarter of sites (*n* = 7/28; 25%) were not sure of the average duration of client engagement.

Notably, three programs (*n* = 3/28; 11%) observed changes in the average length of OAT engagement among their clients post-decriminalization. Two sites (*n* = 2/28; 7%) noted a decrease in the average length of treatment, while one site (*n* = 1/28; 4%) observed an increase.

### Referral pathways and drug alerts

We also examined whether the OAT sites had formal referral pathways (i.e., established collaborations with another organization/service). The majority of sites (*n* = 25/28; 89%) reported having formal referral pathways; most referred clients *to* services (*n* = 22/25; 88%) as well as received referrals *from* services (*n* = 21/25; 84%). OAT sites commonly referred their clients to a range of support programs and/or treatment services (see Table 2). One site (*n* = 1/28; 4%) indicated that the frequency of referrals made to other services after decriminalization had increased.

**Table 2:** Types of services OAT sites reported referring clients to (*n* = 28)

Service type	*n*	%
Residential treatment programs	20	71
Social and family support services	19	68
Inpatient detoxification/withdrawal management programs	18	64
Mental health counseling	18	64
Drug checking services	17	61
Substance use counseling	17	61
Safe consumption services/overdose prevention services	14	50
General practitioners	14	50
Naloxone distribution services	13	46
Outpatient detoxification/withdrawal management programs	12	43
Indigenous-specific services	12	43
Peer support services	10	36
STBBI testing	8	29
Safe supply prescribers	7	25
Other	1	4

The majority of sites (*n* = 22/28; 79%) indicated they were subscribed to either text-based (*n* = 12/22; 54%) or email-based (*n* = 10/22; 45%) toxic drug and health alerts. Among these sites, most (*n* = 18/22; 82%) shared the information contained in drug alerts with their clients.

### Community impacts

Survey respondents were also asked to report on the community impacts of their OAT site. Over half of OAT sites (*n* = 16/28; 57%) experienced police activity either on or near the vicinity of the site. Sites were divided on whether they experienced an increase (*n* = 2/28; 7%) or a decrease (*n* = 2/28; 7%) in police presence since decriminalization. Nine sites (*n* = 9/28; 32%) did not observe any notable changes in law enforcement activity pre- and post-decriminalization.

## Discussion

This study provided an overview of OAT site characteristics in BC and examined any early signals of change related to site operations following the first year of BC’s decriminalization policy, as reported by site staff. The majority of OAT sites reported that, from their perspective, service operations had not changed substantially since the implementation of the policy. This is likely due to the time it takes for policies to effect downstream changes. For instance, drug policy reforms, particularly those as complex as drug decriminalization, take time to influence system-wide operations and societal attitudes before observable changes occur. Research suggests that the effects of drug policy interventions may not manifest until at least two years post-implementation (Olson et al., 2019), while evidence from Portugal, which implemented drug decriminalization over two decades ago, suggests that stigma and broader systemic shifts evolved gradually over time (Gilroy, 2023). It is therefore unsurprising that no major changes to OAT site operations have been observed within the first year, and it will likely take additional time for the policy to realize its short- and long-term goals in this regard.

However, despite the limited operational changes reported by OAT site staff thus far, our study provides several important preliminary insights into early signals of change that may be occurring in the context of decriminalization, highlighting potential considerations for the policy to achieve its intended goals. For instance, while most OAT sites reported serving clients of all ages and genders, staff respondents at two sites indicated a shift in their OAT clientele following the implementation of the policy. One site observed an increase in clients who identify as men, while the other reported an increase in youth clients, particularly those experiencing homelessness. These findings are notable, as men and youth are particularly vulnerable to the impacts of the current overdose crisis. Data suggest that men are more likely than women to die from opioid-related overdoses, with the proportion of men experiencing drug-related harms increasing (Belzak & Halverson, 2018). Youth also face unique challenges in accessing OAT, contributing to lower rates of uptake compared to adults (Giang et al., 2020; Pilarinos et al., 2022). Young people under the age of 30 accounted for 20% of all overdose deaths in BC between 2011 and 2022, highlighting the urgent need for targeted interventions to support this age group (Pilarinos et al., 2022). Barriers such as limited awareness of available services, stigma and concerns about confidentiality, difficulties navigating the healthcare system, and practitioners who are reluctant to initiate OAT for youth can hinder youth from accessing OAT (Pilarinos et al., 2022). Although only a year into decriminalization, these observed shifts to higher-risk clientele such as men and youth may indicate that these individuals may be beginning to feel less stigmatized and more comfortable with accessing treatment services. As decriminalization progresses over time, efforts should continue to be made towards enhancing youth-specific outreach and support services and improving OAT engagement among more vulnerable and younger populations.

Our findings also suggest high and continuous treatment engagement among clients accessing OAT. Specifically, most OAT sites reported serving over 100 clients per month, with high client retention rates, indicating that many clients remain engaged in treatment for six months to more than a year. Moreover, none of the OAT sites reported having a waitlist, and most offered a wide range of OAT medications, suggesting high accessibility of OAT for clients presenting to treatment. Furthermore, three quarters of sites provided OAT take-home doses, three of which indicated there had been an increase in the number of clients receiving take-home doses since decriminalization. However, whether this is attributable specifically to the policy is unclear, particularly given that several changes to prescribing guidelines around the provision of take-home doses have occurred within the same time frame, resulting in a general relaxation of requirements and easier access to take-home doses of these medications (British Columbia Centre on Substance Use (BCCSU), 2023).

Our findings also indicate variability across several measures such as client volume and retention length, with some OAT sites reporting increases and others decreases. In line with take-home doses, these findings also cannot necessarily be attributed to the policy itself and may reflect other simultaneous policy or environmental changes occurring alongside decriminalization. For instance, OAT is known for its cyclical nature, where clients often cycle on and off treatment and may use different OAT medications throughout their lives, which could account for the fluctuations observed (Socías et al., 2020). For sites that experienced an increase in access and retention post-decriminalization, this may suggest that clients are becoming more aware of and comfortable with accessing treatment, possibly due to the reduced stigma associated with decriminalization (Pijl et al., 2022; Socías et al., 2020). Such an outcome would align with the policy’s goals to encourage individuals to initiate and remain engaged in treatment (Ministry of Mental Health & Addictions, 2023). Further, with additional pharmacologic options now available at some sites, such as Sublocade or injectable hydromorphone, as well as risk mitigation prescribing or prescribed “safer” supply programs, which have expanded significantly in recent years and have experienced increased enrollments (Nguyen et al., 2024) (British Columbia Centre on Substance Use (BCCSU) et al., 2022), sites may be seeing an increase in clients who have previously been unsuccessful with other OAT options and are presenting to access these medications.

Conversely, although unlikely given OUD’s high relapse rate (Strang et al., 2020), sites that reported a decrease in uptake and retention might be seeing clients who have achieved their substance use goals and no longer require OAT, or are having their needs met through alternative services, such as residential treatment. Alternatively, this could also signify that sites may be seeing exceptionally transient individuals—which may be aggravated by the current housing crisis—who are more likely to disengage from treatment or may switch between clinics. PWUD who are unhoused may experience specific barriers related to the requirement to demonstrate clinical stability in order to receive OAT take-home doses, which often explicitly outlines stipulations for clients to be housed and/or able to safely store medications (Russell et al., 2024). The burden of daily witnessed dosing may therefore lead to OAT dropout or disengagement. Furthermore, treatment disengagement may be further driven by the toxicity and evolving nature of the drug supply, where clients’ tolerances have increased and OAT dosing may not be sufficient to meet PWUD’s needs (Simpson et al., 2024). It could also mean that some clients are not having their treatment needs fully met and are discontinuing treatment, potentially due to the increasing prevalence of polysubstance use in BC, which has been found to increase the risk of treatment dropout (Crabtree et al., 2020). Indeed, use of both methamphetamine and benzodiazepines is associated with a higher likelihood of OAT dropout (Russell et al., 2023a, b; Franklyn et al., 2017; Papamihali et al., 2021; Eibl et al., 2019). Also, opioid and benzodiazepine withdrawal symptoms overlap in clinical presentation, and it is therefore plausible that clients may be misattributing benzodiazepine withdrawal symptoms to inadequate OAT dosing, leading to treatment discontinuation.

Other factors that may be contributing to fluctuations in clientele volume and engagement in the context of decriminalization include police presence around OAT sites (Greene et al., 2022). For instance, nearly two thirds of OAT sites reported that police activity in the vicinity of their sites was common, of which a majority indicated that police activity either increased or remained at pre-decriminalization levels. Research has shown that the visibility of law enforcement near harm reduction sites can perpetuate fears of surveillance and stigma and deter clients from accessing services (Bardwell et al., 2022). Despite the policy’s aim to shift drug use from a criminal justice issue to a public health issue, ongoing police presence around OAT sites may continue to deter clients from accessing services. As such, to maximize the positive effects of decriminalization, it is crucial to continue to reduce stigma associated with drug use and decrease police presence near sites.

Other noteworthy early signals of change since decriminalization refer to the demand on OAT site staff and resources. Most services reported having multidisciplinary teams of staff and OAT prescribers, consistent with typical harm reduction and treatment settings (de Saxe Zerden et al., 2024). While many site respondents indicated that their current staffing and resources generally meet their clients’ needs, some noted that the demand on their site’s staff had increased since decriminalization. Specifically, sites reported increases in workload, administrative tasks, and training and development needs, as well as client interactions. Moreover, several sites expressed concerns about being increasingly under-resourced, particularly in areas such as medical and harm reduction supplies, medication, technological equipment, educational materials, and general space and infrastructure. While more comprehensive data are needed to assess whether decriminalization has contributed to these perceived changes in demand, these findings suggest that sites may already be operating at or near capacity. Literature demonstrates that harm reduction and treatment staff have experienced considerable burnout and traumatic stress in the context of the ongoing overdose crisis (Taha et al., 2022). Burnout among staff contributes to high turnover rates, which can destabilize service delivery, disrupt client care, reduce the overall quality of service, and lead to gaps in the continuity of care (Taha et al., 2022). Decriminalization aims to increase treatment uptake; however, for decriminalization to successfully achieve this goal, the corresponding increase in treatment demand must be met by adequate resources. This means investments in staffing and resources need to be scaled up appropriately to match the anticipated rise in client engagement if the policy is to meet its goals. Failure to do so could lead to overwhelmed services, longer wait times, reduced quality of care, and ultimately a failure to retain clients in treatment.

Crucially, the effectiveness of OAT relies on the seamless integration and connection between various service types and programs (Pijl et al., 2022). Our findings indicate that OAT programs in BC are actively working towards these goals by ensuring that their services are integrated with and/or affiliated with other essential health and harm reduction services, underscoring the utility of OAT as a key component of the continuum of care for PWUD in the province. Most OAT sites reported that they regularly refer clients to a wide range of external resources and services, including residential treatment programs, social and family support services, withdrawal management (detox), and drug checking services, as well as mental health and substance use–related counseling. This comprehensive referral network not only enhances the accessibility of these critical services but also supports a holistic approach to care that addresses the complex and multifaceted needs of PWUD. Furthermore, most OAT sites indicated that they actively subscribe to and disseminate toxic drug and health alerts within their communities. This practice highlights the role of OAT programs in not only providing direct treatment but also in connecting PWUD with crucial information that can help mitigate harms associated with drug use. Research has shown that PWUD often modify their behaviour to adopt safer practices after receiving a drug alert, which can significantly reduce the risk of overdose (Daowd et al., 2023). Improving drug alerts to reach smaller communities using real-time information from drug-checking programs based on testing from local drug supplies could further reduce this risk.

Regardless of OAT sites’ efforts as part of a comprehensive approach to addressing drug use and related harms, in the specific context of decriminalization, only three sites received formal decriminalization training, yet many suggested their site and staff would benefit from this. Thus, more can be done to bolster the decriminalization policy and its ability to increase treatment uptake and engagement among PWUD, and ultimately reach its goals. This includes the need for increased training of OAT site staff, messaging and awareness around the policy, and reducing potential barriers that deter PWUD from seeking treatment.

### Strengths and limitations

This study has several strengths, including a high response rate and wide geographical distribution of site respondents, including responses inclusive of all five Health Authorities and from both rural and urban communities. However, despite our best efforts, we cannot be certain that we identified every OAT site in the province that met our inclusion criteria, and some sites may have been missed.

Moreover, self-reported surveys are prone to several biases, including selection, recall, and response biases, impacting data reliability (Waters et al., 2022). Specifically, the results presented in our study reflect OAT site staff’s individual perceptions of their site’s operations and clientele characteristics. Although over two thirds of sites indicated they systematically collect and record clientele socio-demographic data, which strengthens the validity of the responses to these questions, respondents were not explicitly asked to consult their databases and may be reporting on their impressions of survey indicators rather than objective data. In addition, despite the high response rate, the total sample size was low, which may hinder the generalizability of our results.

Further, although our questions were operationalized to elicit specific effects of decriminalization on each survey measure, we cannot guarantee accurate interpretation of the questions, and some responses were also skipped by participants, resulting in missing data. Different types and roles of respondents may have also influenced their perceptions, for instance, if they were more or less knowledgeable about a particular operational aspect of their site. Responses also need to be considered in the context of other potential confounding factors, and individual sites may have experienced impacts related to decriminalization that are not necessarily applicable to other sites. Specifically, there is a broad spectrum of community and local acceptance and/or approaches towards harm reduction and public health–oriented initiatives, and local responses to drug policies reflect a complex interplay of political, social, and cultural factors that may influence the uptake of certain programs. Additionally, evaluating drug policies presents inherent challenges, particularly in isolating the specific impacts of decriminalization on OAT services (Schuler et al., 2021). The drug policy landscape is complex and dynamic, where multiple overlapping policies and initiatives have been introduced or adapted concurrently, such as risk mitigation prescribing from the COVID-19 era (British Columbia Centre on Substance Use (BCCSU) et al., 2022) and safer supply programs (Nguyen et al., 2024). These, particularly in the absence of any control, complicate the ability to tease apart the direct impacts of decriminalization. We also excluded youth-specific sites, as well as broader primary and community health settings that offer OAT, which may have experienced differential impacts and should be explored in future research.

Additional future research should explore potential differences between sites located in urban and rural areas, individual/treatment-level changes (e.g., impacts of decriminalization on OAT engagement/retention patterns across specific OAT medication types), as well as site funding, including the extent to which operating budgets may have changed since decriminalization, impacting site staffing and resource demand.

## Conclusion

This study characterized OAT site operations across BC, offering insights into early indicators of operational changes following the first year of decriminalization, based on the perceptions of OAT site staff. Our results largely indicate that the policy has had minimal impacts on OAT site operations, including in relation to clientele socio-demographics, uptake and engagement, referral pathways, funding sources and investments, and community impacts. However, some sites experienced an increase in staffing and resource demand since the implementation of the policy. Responses suggest the need for increased staff training and continued investments into OAT to better support the anticipated demand on services if decriminalization is to reach its goal of improving access to treatment among PWUD.

## Contributions to knowledge

What does this study add to existing knowledge?

- This study describes the current landscape of OAT program operations in BC, filling a critical gap in the literature.
- The study also offers a snapshot of early signals of operational changes that OAT sites may be experiencing since the introduction of the policy. As such, this study provides a baseline for which to monitor how or whether the decriminalization policy may be reaching its short- and long-term goals of increasing PWUD engagement in treatment.

What are the key implications for public health interventions, practice, or policy?

- The results from this study offer baseline data that can be used to monitor operational OAT trends going forward.
- Results highlight minimal changes in OAT operations and programming across BC within the first year of the policy’s implementation. However, results suggest preliminary signals of change related to increased demand on OAT staff and resources, as well as potential shifts to more vulnerable clientele.
- These findings indicate that in order for decriminalization to realize its goal of increasing treatment engagement and retention among PWUD, OAT programs may require additional funding and investments. Additional monitoring of the policy’s specific impacts and more comprehensive data are needed to draw robust conclusions.

## Data Availability

The data that support the findings of this study are available from the corresponding author upon reasonable request.
